# Descriptive analysis of prescription interception patterns: characterizing medication safety risks in an outpatient setting

**DOI:** 10.3389/fphar.2026.1826012

**Published:** 2026-07-14

**Authors:** Ning Li, Weiwei Tang, Nan Li, Chang Gong, Qiuhong Wei, Jiawei Gong, Jinghui Zhai, Sixi Zhang

**Affiliations:** 1 Department of Clinical Pharmacy, The First Hospital of Jilin University, Changchun, Jilin, China; 2 School of Pharmaceutical Science, Jilin University, Changchun, Jilin, China

**Keywords:** departmental variation, electronic prescribing, forced interception, medication safety, targeted interventions

## Abstract

**Introduction:**

Prescription errors remain a significant challenge to medication safety in hospital settings. Forced Interception (FI) systems, which automatically flag and block potentially problematic prescriptions, serve as critical safeguards against adverse drug events. However, the specific characteristics and underlying causes of intercepted prescriptions, particularly in Chinese hospital contexts, require further investigation to inform targeted quality improvement strategies. This study aimed to analyze the characteristics and interception reasons of FI prescriptions in a hospital setting, with the goal of identifying patterns that could guide system upgrades, clinical training, and policy interventions.

**Methods:**

This study conducted a retrospective analysis of FI prescriptions intercepted by the hospital’s electronic prescribing system. Prescriptions were analyzed for interception reasons, drug categories, specific medications, and prescribing department patterns. Data were collected and categorized to identify the most frequent issues and drug types involved in forced interceptions.

**Results:**

A total of FI prescriptions were analyzed. The most common interception reasons were "Treatment duration exceeded" (54.89%) and "Exceeding Dosage" (28.11%), together accounting for the majority of interceptions. Traditional Chinese medicine and central nervous system drugs were the most frequently intercepted drug categories. The top three intercepted medications were Duloxetine Hydrochloride Enteric Capsules (3.63%), Atorvastatin Calcium Tablets (3.11%), and Tandospirone Citrate Capsules (2.81%). Departmental analysis revealed distinct prescribing patterns: the cardiology department showed high interceptions for hyperlipidemia-related drugs, while the mental health department had numerous interceptions for long-term antidepressant and anxiolytic prescriptions.

**Discussion:**

The study suggests that targeted interventions, including upgrading electronic prescribing systems, department-specific training, and forming special review panels, are necessary to reduce prescription errors and improve medication safety. Future work should focus on multicenter studies and, as a longer-term direction, explore the potential of artificial intelligence for dynamic risk prediction to enhance the continuous optimization of medical quality.

## 1 Introduction

In recent years, the use of Intelligent Management System for clinical rational drug use (IMSD) has become increasingly prevalent in healthcare settings, particularly in hospitals (Alqahtani et al., 2025). These systems aim to enhance patient safety and improve the quality of care by providing real-time alerts and recommendations to healthcare providers. One critical application of IMSD is the implementation of Forced Interception (FI), which prevent physicians from prescribing medications or therapies that are deemed inappropriate or potentially harmful based on predefined clinical guidelines or rules (Martin and Létinier, 2026). While FI are designed to mitigate medical errors and ensure adherence to best practices, they also generate valuable datasets that reflect deviations from standard care pathways (Ong et al., 2025). These datasets offer insights into prescribing behaviors, potential knowledge gaps, and systemic issues within clinical practice.

IMSD have become integral to modern medication safety strategies, yet their effectiveness varies significantly across healthcare contexts. Globally, medication errors contribute to substantial patient harm, prompting the World Health Organization (WHO) to launch the Third Global Patient Safety Challenge: Medication Without Harm (2017), which explicitly advocates for electronic prescribing systems as a core intervention to reduce avoidable medication-related harm by 50% (Donaldson et al., 2017; Sousa et al., 2025). This initiative aligns with the Institute for Safe Medication Practices (ISMP) ‘Key Elements of the Medication Use System’ framework, which identifies electronic decision support, standardized order sets, and error surveillance as critical leverage points. While systematic reviews demonstrate that well-implemented IMSD can reduce prescription errors by 30%–80%, these benefits are moderated by local implementation factors, prescriber experience, and institutional safety culture (Eze et al., 2026; Devin et al., 2020). Most existing studies originate from large, academic medical centers in high-income countries, creating a critical evidence gap regarding how local prescribing culture, institutional rule architectures, and population-specific factors influence IMSD effectiveness in single-center or resource-variable settings (Mummadi and Mishra, 2018).

Despite the widespread adoption of IMSD, relatively little research has been conducted to systematically analyze the patterns and causes of intercepted prescriptions. Understanding the reasons behind these intercepts is essential for improving clinical decision-making, refining IMSD algorithms, and ultimately enhancing patient outcomes. In this study, we aim to analyze FI prescriptions from our hospital to identify prescribing issues and their underlying causes. By examining the characteristics of these intercepted prescriptions, we seek to provide actionable insights for clinicians and healthcare institutions. Additionally, we aim to highlight the importance of continuous education and system optimization in reducing prescribing errors and improving patient safety. This study not only contributes to the understanding of prescribing behaviors but also offers practical recommendations for enhancing the effectiveness of IMSD in clinical practice.

## 2 Materials and methods

### 2.1 Rule settings of the IMSD

Based on the IMSD (vendor: Tianji Health Medical Technology Co., LTD., version: V2.1, integration: embedded in Electronic Medical Record system HISystem V6.0, launched 2024), the relevant rational drug used intervention rules were set using the built-in maintenance tools of the system. The prescription review rules were formulated in accordance with the drug instructions, covering aspects such as indications, contraindications, precautions, dosage and administration, drug interactions, special population medication, and drug allergies. The FI in the rule settings was a hard-stop alert requiring prescriber correction before order completion, distinct from soft alerts that allowed order continuation with justification. The system directly prevented physicians from writing incorrect prescription orders. The prescriptions could only be reviewed and approved after the physicians had modified them to be correct. The knowledge based was regularly updated and maintained to ensure its consistency with the latest clinical evidence.

### 2.2 Data collection

This was a retrospective study. No specific inclusion/exclusion criteria were used, as the system processed all prescriptions based on the predefined rules. During the 2024 study period, the prescription review system reviewed a total of 2,295,807 outpatient prescriptions. Prescription information was extracted from the FI database, which intercepted a total of 53,259 prescriptions from January through December 2024, ensuring complete data coverage throughout the study period. All experimental protocols were approved by Ethics Committee of the First Hospital of Jilin University.

### 2.3 Statistical methods

Data were entered using Excel software, and statistical analysis of the effective data before and after the refinement of the rules was conducted using both Excel and SPSS 27.0 software. Chi-square tests for categorical comparisons, with significance set at p < 0.05.

## 3 Results

### 3.1 General of characteristics in FI prescriptions

During the 2024 study period, the prescription review system reviewed a total of 2,295,807 outpatient prescriptions. Of these, 53,259 prescriptions (2.3%) were intercepted and extracted from the FI database from January through December 2024, ensuring complete data coverage throughout the study period.

In terms of problems with FI prescriptions, “Treatment duration exceeded” (29,233, 54.89%) and “Exceeding Dosage” (14,973, 28.11%) were the most common, followed by “Other Inappropriate Medication Use” (5,673, 10.65%), “Inappropriate Administration Method” (1,540, 2.89%), and “Duplicate Medication” (1,116, 2.1%).

Regarding the types of medicines involved in FI prescriptions, Chinese Patent Medicine (proprietary traditional Chinese medicine preparations approved by the National Medical Products Administration) accounted for the largest proportion (2,945, 19.67%), followed by Central Nervous System Medicine (2,559, 17.09%), Electrolyte Preparations (2,487, 16.61%), Anti - Infective Medicine (1,751, 11.69%), and Circulatory System Medicine (936, 6.25%).

The Department of Internal Medicine had the highest number of FI prescriptions (23,619, 44.37%), followed by Surgery (6,971, 13.07%), the Mental Health Department (5,706, 10.71%), Pediatrics (4,308, 8.08%), and Obstetrics (1,006, 1.88%).

In terms of diagnoses associated with FI prescriptions, Hypertension was the most common (6,013, 11.29%), followed by Anxiety - depressive state (5,252, 9.86%), Hyperlipidemia (4,669, 8.77%), Diabetes Mellitus (4,047, 7.6%), and General medical examination (3,696, 6.94%) (Tables 1, 2).

**TABLE 1:** General of characteristics in FI prescriptions.

General characteristics	Count	Proportion(%)
Problem		
Treatment duration exceeded	29233	54.89
Exceeding dosage	14973	28.11
Other inappropriate medication use	5673	10.65
Inappropriate administration method	1540	2.89
Duplicate medication	1116	2.10
Medicine type		
Chinese patent medicine	2945	19.67
Central nervous system medicine	2559	17.09
Electrolyte preparations	2487	16.61
Anti-infective medicine	1751	11.69
Circulatory system medicine	936	6.25
Ophthalmic medicine	709	4.74
Endocrine medicine	502	3.35
Digestive system medicine	437	2.92
Respiratory system medicine	362	2.42
Blood medicine	306	2.04
Departments		
Internal medicine	23619	44.37
Surgery	6971	13.07
Mental health department	5706	10.71
Pediatrics	4308	8.08
Obstetrics	1006	1.88
Others	11649	22.16
Diagnosis		
Hypertension	6013	11.29
Anxiety-depressive state	5252	9.86
Hyperlipidemia	4669	8.77
Diabetes mellitus	4047	7.60
General medical examination	3696	6.94
Insomnia	3394	6.37
Osteoporosis	2180	4.09
Arthritis	2157	4.05
Coronary atherosclerotic heart disease	1987	3.73
Cerebral infarction	1722	3.23

Data from a tertiary hospital in Changchun, China.

**TABLE 2:** Top 10 medications in FI prescriptions.

Medicine	Count	Proportion(%)
Duloxetine hydrochloride enteric capsules	1933	3.63
Atorvastatin calcium tablets	1658	3.11
Tandospirone citrate capsules	1496	2.81
Clopidogrel hydrogen sulphate tablets	737	1.38
Vitamin D drops	1136	2.13
Entecavir tablets	1048	1.97
Levofloxacin eye drops	1015	1.91
Dexzopiclone tablets	765	1.44
Bupropion hydrochloride sustained-release tablets	756	1.42
Flurbiprofen gel patch	613	1.15

Data from a tertiary hospital in Changchun, China.

### 3.2 Analysis of top ten interceptions in FI prescriptions

The analysis of the top ten medications FI by IMSD revealed the following distribution: Duloxetine Hydrochloride Enteric Capsules were the most frequently intercepted, accounting for 3.63% (n = 1,933) of all interceptions. This was followed by Atorvastatin Calcium Tablets at 3.11% (n = 1,658) and Tandospirone Citrate Capsules at 2.81% (n = 1,496). Vitamin D Drops represented 2.13% (n = 1,136), while Clopidogrel Hydrogen Sulphate Tablets, Entecavir Tablets, and Levofloxacin Eye Drops constituted 1.38% (n = 737), 1.97% (n = 1,048), and 1.91% (n = 1,015), respectively. Dexzopiclone Tablets, Bupropion Hydrochloride Sustained-Release Tablets, and Flurbiprofen Gel Patch accounted for smaller proportions, ranging from 1.15% to 1.44% (n = 613–765). These findings highlight the specific medications most commonly flagged by the system, with Duloxetine Hydrochloride Enteric Capsules, Atorvastatin Calcium Tablets, and Tandospirone Citrate Capsules being the top three contributors to prescription interceptions.

### 3.3 Analysis of departments’ FI prescriptions specific situations

“Medication beyond the treatment course” and “medication beyond the prescribed dosage” were the top two interception reasons across all departments. The Department of Cardiovascular Medicine had the highest number for “medication beyond the treatment course” (2,265, 74.29%), followed by Endocrinology and Metabolism (1,758, 72.76%), Mental Health (4,119, 72.19%), Neurology (3,479, 62.25%), and Nephrology (3,107, 58.60%). For “medication beyond the prescribed dosage”, Neurology led with 1,377 (24.64%), followed by Cardiovascular Medicine (364, 11.94%), Endocrinology and Metabolism (264, 10.93%), Mental Health (480, 8.41%), and Nephrology (297, 5.60%) (Tables 3, 4).

**TABLE 3:** Departments’ FI prescriptions specific situations.

Department	Category	Description	Count	Proportion
Department of mental health	Issues	Medication beyond the treatment course	4119	72.19%
		Medication beyond the prescribed dosage	480	8.41%
	Drugs	Duloxetine hydrochloride enteric-coated capsules	1770	31.02%
		Tandospirone citrate capsules	1444	25.31%
		Bupropion hydrochloride sustained-release tablets	755	13.23%
	Diagnosis	Anxiety and depression state	4458	78.13%
		Mental disorder	557	9.76%
		Sleep disorder	426	7.47%
Department of neurology	Issues	Medication beyond the treatment course	3479	62.25%
		Medication beyond the prescribed dosage	1377	24.64%
	Drugs	Pregabalin capsules	277	4.96%
		Alendronate sodium tablets	247	4.42%
		Pyridostigmine bromide tablets	246	4.40%
	Diagnosis	Myasthenia gravis	1036	18.54%
		Insomnia	955	17.09%
		Osteoporosis	702	12.56%
Department of nephrology	Issues	Medication beyond the treatment course	3107	58.60%
		Medication beyond the prescribed dosage	297	5.60%
	Drugs	Atorvastatin calcium tablets	328	8.38%
		Vitamin D drops	304	7.77%
		Shenyanling granules	236	6.03%
	Diagnosis	Nephrotic syndrome	1047	26.75%
		Hypertension	1009	25.78%
		Renal insufficiency	648	16.56%
Department of cardiovascular medicine	Issues	Medication beyond the treatment course	2265	74.3%
		Medication beyond the prescribed dosage	364	11.90%
	Drugs	Clopidogrel tablets	667	21.88%
		Atorvastatin calcium tablets	475	15.60%
		Dagliazine tablets	182	6.00%
	Diagnosis	Hyperlipidemia	1433	47.31%
		Hypertension	913	30.14%
		Coronary atherosclerotic heart disease	571	18.85%
Department of endocrinology and metabolism	Issues	Medication beyond the treatment course	1758	72.76%
		Medication beyond the prescribed dosage	264	10.93%
	Drugs	Atorvastatin calcium tablets	318	13.16%
		Insulin degludec and insulin aspart injection	258	10.68%
		Sitagliptin and metformin tablets	175	7.24%
	Diagnosis	Diabetes mellitus	1476	61.09%
		Hyperlipidemia	743	30.75%
		Hypertension	356	14.74%

Data from a tertiary hospital in Changchun, China.

**TABLE 4:** Distribution of medication problem types by department.

Deparment	Medication beyond the treatment course	Medication beyond the prescribed dosage	χ^2^	P
Department of mental health	4119	480	723.474	P < 0.0001
Department of neurology	3479	1377		
Department of nephrology	3107	297		
Department of cardiovascular medicine	2265	364		
Department of endocrinology and metabolism	1758	264		

Data from a tertiary hospital in Changchun, China.

**FIGURE 1:**
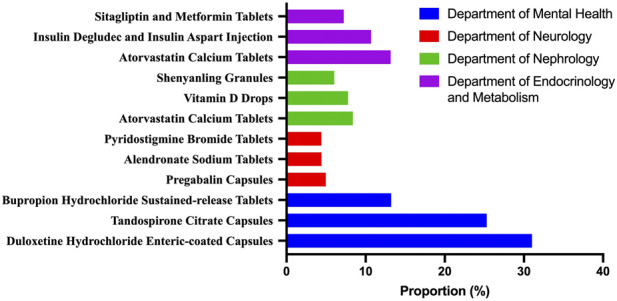
Proportion of top three intercepted medications by clinical department.

The FI prescriptions involved various drugs (Figure 1). In Mental Health, the main intercepted drugs were Duloxetine Hydrochloride Enteric - coated Capsules (1,770, 31.02%), Tandospirone Citrate Capsules (1,444, 25.31%), and Bupropion Hydrochloride Sustained - release Tablets (755, 13.23%). For Neurology, the primary intercepted drugs were Pregabalin Capsules (277, 4.96%), Alendronate Sodium Tablets (247, 4.42%), and Pyridostigmine Bromide Tablets (246, 4.40%). In Nephrology, the main intercepted drugs were Atorvastatin Calcium Tablets (328, 8.38%), Vitamin D Drops (304, 7.77%), and Shenyanling Granules (236, 6.03%). Cardiovascular Medicine mainly intercepted Clopidogrel tablets (667, 21.88%), Atorvastatin Calcium Tablets (475, 15.58%), and Dagliazine Tablets (182, 5.97%). Endocrinology and Metabolism primarily intercepted Atorvastatin Calcium Tablets (318, 13.16%), Insulin Degludec and Insulin Aspart Injection (258, 10.68%), and Sitagliptin and Metformin Tablets (175, 7.24%).

Different departments had distinct main intercepted diagnoses. Mental Health mainly intercepted prescriptions for “Anxiety and depression state” (4,458, 78.13%). Neurology focused on “Myasthenia gravis” (1,036, 18.54%), “Insomnia” (955, 17.09%), and “Osteoporosis” (702, 12.56%). Nephrology primarily intercepted prescriptions for “Nephrotic syndrome” (1,047, 26.75%), “Hypertension” (1,009, 25.78%), and “Renal insufficiency” (648, 16.56%). Cardiovascular Medicine mainly intercepted prescriptions for “Hyperlipidemia” (1,433, 47.31%), “Hypertension” (913, 30.14%), and “Coronary atherosclerotic heart disease” (571, 18.85%). Endocrinology and Metabolism mostly intercepted prescriptions for “Diabetes mellitus” (1,476, 61.09%), “Hyperlipidemia” (743, 30.75%), and “Hypertension” (356, 14.74%).

This analysis of FI prescriptions shows that “medication beyond the treatment course” and “medication beyond the prescribed dosage” were the most common interception reasons across departments. Each department had distinct patterns in intercepted drugs and diagnoses, indicating the need for targeted interventions to reduce prescription errors and improve patient safety.

## 4 Discussion

A IMSD relies heavily on robust software support, with a powerful drug rule database being the key to effective pre-dispensing pharmaceutical management. To enhance prescription intervention quality, medical institutions should establish a proprietary, customizable knowledge base that aligns with their clinical and operational realities, allowing for well-founded and efficient pre-dispensing review processes. It is essential to clarify the governance framework underlying the IMSD’s interception mechanism. The system is explicitly positioned as an augmented decision-support tool, not an autonomous hard-stop system. Real-time interception is strictly confined to explicit, high-risk errors that require no clinical judgment—specifically, validated absolute contraindications. For complex scenarios or cases where evidence remains controversial, the system provides risk stratification alerts rather than blocking the prescription, and the final decision authority rests with the prescribing physician and reviewing pharmacist. Every intercepted prescription is logged, subjected to periodic retrospective review, and reported back to the relevant clinical departments. This human-in-the-loop architecture ensures transparent clinical accountability, preserves professional autonomy in nuanced clinical judgments, and aligns with contemporary standards for responsible clinical decision support.

This positioning is consistent with the World Medical Association’s emphasis on “augmented intelligence”—defined as AI designed to support, not replace, human capabilities in healthcare—and its Physician-in-the-Loop (PITL) principle, which mandates that licensed physicians retain final authority and ultimate clinical responsibility over all AI-influenced decisions (Olawade et al., 2026). Similarly, the American Medical Association has adopted “augmented intelligence” as its preferred framing, stressing that physicians must remain at the forefront of AI adoption, actively reviewing and validating AI outputs to ensure that clinical accuracy and patient safety are maintained (Nature, 2025) (For trustworthy AI, keep the human in the loop, 2025). Across ethical, legal, and practical domains, authoritative consensus holds that AI must function as an assistive tool, with final clinical accountability resting with qualified healthcare professionals-a principle that our institutional workflow embeds at every level (Ong et al., 2026).

Yet translating these principles into sustained clinical practice presents tangible challenges. Recent evidence underscores that AI-assisted medication management achieves optimal outcomes when clinicians are active partners in system governance rather than passive recipients of alerts. Spanakis et al. (2026) propose a “closing the loop” framework for drug-drug interaction management, in which clinician feedback is structurally incorporated into model learning—moving beyond post hoc validation to continuous, bidirectional knowledge refinement (Spanakis et al., 2026). This aligns with our institutional practice of periodic retrospective review, though formalizing real-time learning loops remains an area for development. Olawade et al. (2026) identify implementation barriers directly affecting clinician experience, including workflow integration difficulties, regulatory uncertainties for adaptive systems, and the need for adaptive training protocols to maintain user competency (Olawade et al., 2026). Their finding that clinician trust is contingent upon transparent collaboration models resonates with our observation that prescriber acceptance of interception varies by clinical context. Notably, Zhang et al. (2025) demonstrate that AI-assisted quality control circles led by clinical pharmacists increased rational PPI use from 66.51% to 93.43%, highlighting that pharmacist-led human-AI collaboration yields superior outcomes compared to automated alerts alone (Zhang et al., 2025). This evidence supports our recommendation to expand clinical pharmacist involvement and reinforces that IMSD optimization must prioritize clinician enablement alongside technical refinement. The tension between system automation and professional autonomy—central to modern debates on AI in healthcare—requires ongoing negotiation at the institutional level, with clear accountability frameworks preserving human responsibility for final clinical judgments.

While dose and duration errors may appear as expected findings in a rule-based IMSD, their interpretation extends beyond surface-level prescription correction. Prescription interception serves not merely as a technical gatekeeping function, but as a catalyst for multidimensional healthcare improvement. By preventing inappropriate dosing and excessive duration, the system directly reduces per-visit drug expenditure for both outpatients and inpatients, aligning with national healthcare performance metrics. Furthermore, interception corrects patient-driven demands for unnecessary medications, addressing over-prescribing and drug misuse that persist as entrenched clinical habits rather than evidence-based practice. Certain dose-duration combinations reflect system inertia and outdated prescribing patterns despite updated guidelines—for instance, extended antibiotic courses or fixed-dose combinations for chronic conditions that persist as “standard practice” despite recommendations for shorter durations or individualized titration. Curtailing excessive prescribing not only reduces pharmaceutical waste but also prevents downstream costs associated with adverse drug reactions and their subsequent treatments. Optimized regimens can shorten treatment courses, while the iterative feedback loop enhances prescriber awareness of rational drug use beyond isolated error correction. These findings represent actionable intelligence for system-level redesign, such as refining existing rule-based alerts for evolving guidelines, rather than mere frequency tabulation.

The generalizability of our findings is inherently bounded by the regulatory and institutional ecology within which this study was conducted. The FI rates and error patterns reported here are not simply reflections of prescriber behavior in the abstract, but are actively co-produced by three layers of local context. First, institutional rule architecture: our IMSD’s drug rule database embeds specific dosage thresholds, duration limits, and interaction criteria that differ across hospitals; what triggers interception here may pass unchecked elsewhere, or vice versa. Second, regulatory framework embedding: national and regional healthcare policies—including reimbursement rules, antimicrobial stewardship mandates, and traditional Chinese medicine practice guidelines—shape prescribing options available to clinicians in ways that vary geographically and temporally. For example, the high interception rate for traditional Chinese medicine (19.67%) partly reflects ongoing national efforts to standardize TCM usage within evidence-based frameworks, a context-specific priority that may not parallel other healthcare systems. Third, clinical workflow configuration: our CPOE system’s integration with laboratory reporting, the availability of clinical pharmacist consultation, and department-specific prescribing protocols create a unique decision-making environment. Consequently, we caution against direct extrapolation of our FI prevalence figures or departmental rankings to other centers. Instead, we propose that our value lies in demonstrating a replicable analytical framework for interrogating local prescribing-system interactions, and in identifying categories of evidence-practice gaps (e.g., duration management in chronic disease, individualized dosing for psychotropics) that likely resonate across contexts even if their specific manifestations differ.

This study analyzed FI prescriptions data to uncover core medication-use issues, common drug characteristics, and differences among departments. “Treatment duration exceeded” (54.89%) and “Exceeding Dosage” (28.11%) are the top two reasons for FI prescriptions, consistent with previous studies (Alkanj et al., 2025). Doctors often extend treatment duration due to the ongoing nature of chronic conditions like hypertension and diabetes, while insufficient patients follow-up slows dosage adjustments. Traditional Chinese medicine (19.67%) and central nervous system drugs (17.09%) were frequently intercepted, suggesting variability in evidence-based dosing guidelines. The complex ingredients and unclear evaluation standards of traditional Chinese medicine may result in excessive duration of use, whereas central nervous system drugs (e.g., antidepressants), given their long-term treatment requirements, are harder to manage in terms of duration. The top three intercepted drugs are Duloxetine Hydrochloride Enteric Capsules (3.63%), Atorvastatin Calcium Tablets (3.11%), and Tandospirone Citrate Capsules (2.81%)-share the common characteristics of requiring individualized dosage and duration adjustments. Duloxetine, an SNRI, can cause withdrawal reactions if stopped suddenly, and long-term use may increase tolerance risk (Migliorini et al., 2023; Şahan and Parlakkaya Yıldız, 2019). Atorvastatin dosage should be based on lipid levels (Maheta et al., 2025; Arsenault et al., 2014), yet doctors may prescribe fixed doses, leading to frequent interceptions. Tandospirone citrate for anxiety requires balance short-term symptom control with long-term dependence risk, highlighting the need for multidisciplinary duration assessment in mental health. These high-FI drugs indicate a need for better individualized guidance and systematic review, including priority monitoring lists and potentially, in future implementations, real-time dosage recommendation tools integrated with clinical decision support systems.

The departmental distribution reveals clinically meaningful patterns. In cardiology, hyperlipidemia diagnoses (47.31%) closely linked to atorvastatin interceptions (15.58%) (Rubino et al., 2021). Given the non-linear dose-efficacy relationship of statins (Pedersen, 2010), some doctors aiming for rapid targets choose high doses while overlooking liver enzyme monitoring and myopathy risks (Stone et al., 2014; Kunakorntham et al., 2022; Kido et al., 2015). Strengthening the “start-low, adjust-gradually” principle through professional training and using electronic systems to automatically link lipid reports with prescription suggestions is warranted. In the mental health, “long-term prescriptions” accounted for interceptions (72.19%), mainly for antidepressant/anxiolytic drugs (duloxetine and tandospirone), with diagnoses focused on anxious and depressive states (78.13%). The conflict between long-term maintenance of psychotropic drugs and patients’ fluctuating symptoms is likely the primary driver (Yee et al., 2019). A “staged efficacy evaluation” mechanism, using scale scores and biochemical indicators to determine the necessity of extending drug periods, is advised. In the nephrology department, vitamin D drop prescriptions are intercepted at 7.77%, linked to nephrotic syndrome diagnoses (26.75%), reflecting the complexity of calcium-phosphorus metabolism management in chronic kidney disease (Gembillo et al., 2021; Jørgensen et al., 2024). Dynamic adjustment based on blood calcium and phosphorus levels is essential, and clinical pharmacists should join multi-disciplinary management teams to provide reasonable drug adjustment plans.

Based on these findings, we propose several actionable strategies. First, upgrade electronic prescribing systems by integrating patients’ historical medication data, laboratory results, and guideline-recommended plans to enable automated review of duration and dosage (Tantray et al., 2024). Second, conduct department-specific medication safety training focusing on traditional Chinese medicine, psychotropic drugs, and chronic disease management, using case-based scenario simulation. Third, form a “High-Alert Drug Special Review Panel” to analyze the root causes of the top 10% of intercepted drugs and provide periodic feedback to clinical departments. Fourth, drive the development of regional healthcare information platforms to share medication records across institutions, reducing duplicate prescriptions and drug interaction risks.

We acknowledge several limitations. First, this study is descriptive in nature and evaluates intercepted prescriptions only; it does not assess the diagnostic performance, sensitivity, specificity, or clinical appropriateness of the interception system itself. We cannot determine whether the system failed to intercept genuinely inappropriate prescriptions (false negatives) or whether intercepted prescriptions would have resulted in actual patient harm if dispensed. Second, as a single-center study, our findings inherently reflect the local prescribing culture, institutional rule settings, and specific patient population characteristics of our institution. The generalizability may be constrained by contextual factors unique to our healthcare environment, including formulary restrictions, electronic health record configurations, and specialty referral patterns. Potential sources of bias include volume-based differences, where high-volume prescribers may exhibit different prescribing patterns due to increased experience or workflow efficiencies, and prescriber experience variation, where clinicians at different career stages or training backgrounds may approach therapeutic decisions divergently. Future multicenter studies are needed to validate our findings across diverse institutional contexts and quantify the impact of site-specific variables. Additionally, the integration of artificial intelligence—such as machine learning models for adverse drug event prediction—represents a potential future direction that could enable dynamic risk prediction models and advance from retrospective identification to real-time quality improvement. However, such AI-augmented approaches were not evaluated in the present study and remain speculative at this stage.

## 5 Conclusion

This study demonstrates that IMSD-based pre-dispensing review effectively identifies prevalent medication risks, particularly duration and dosage errors, with notable department-specific and drug-specific patterns. These findings highlight actionable targets for localized intervention, though the impact of integrated technological, educational, and policy responses requires prospective validation. Future research directions may include exploring dynamic, AI-augmented closed-loop systems; however, such approaches extend beyond the scope of the present descriptive analysis and would require dedicated evaluation in subsequent studies.

## Data Availability

The raw data supporting the conclusions of this article will be made available by the authors, without undue reservation.

## References

[B1] Alkanj A. Godet J. Johns E. Gourieux B. Michel B. (2025). Deep learning classification of drug-related problems from pharmaceutical interventions issued by hospital clinical pharmacists during medication prescription review: a large-scale descriptive retrospective study in a French university hospital. Eur. J. Hosp. Pharm. 32, 324–328. 10.1136/ejhpharm-2024-004139 39122480

[B2] Alqahtani S. S. Menachery S. J. Alshahrani A. Albalkhi B. Alshayban D. Iqbal M. Z. (2025). Artificial intelligence in clinical pharmacy-A systematic review of current scenario and future perspectives. Digit. Health 11, 20552076251388145. 10.1177/20552076251388145 41146680 PMC12553886

[B3] Arsenault B. J. Barter P. DeMicco D. A. Bao W. Preston G. M. LaRosa J. C. (2014). Prediction of cardiovascular events in statin-treated stable coronary patients of the treating to new targets randomized controlled trial by lipid and non-lipid biomarkers. PLoS One 9, e114519. 10.1371/journal.pone.0114519 25531109 PMC4273994

[B4] Devin J. Cleary B. J. Cullinan S. (2020). The impact of health information technology on prescribing errors in hospitals: a systematic review and behaviour change technique analysis. Syst. Rev. 9, 275. 10.1186/s13643-020-01510-7 33272315 PMC7716445

[B5] Donaldson L. J. Kelley E. T. Dhingra-Kumar N. Kieny M.-P. Sheikh A. (2017). Medication without harm: WHO’s third global patient safety challenge. Lancet 389, 1680–1681. 10.1016/S0140-6736(17)31047-4 28463129

[B6] Eze A. U. Marufu T. Amagyei A. Nelson D. Laparidou D. C Manning J. (2026). Assessing the effectiveness of interventions implemented by nurses to reduce medication administration errors in hospitalised acute adult patient settings: systematic review and meta-analysis. J. Clin. Nurs. 35, 1104–1124. 10.1111/jocn.70109 41031474 PMC12862564

[B7] Gembillo G. Siligato R. Amatruda M. Conti G. Santoro D. (2021). Vitamin D and Glomerulonephritis. Med. (Kaunas) 57, 186. 10.3390/medicina57020186 33671780 PMC7926883

[B8] Jørgensen H. S. de Loor H. Billen J. Peersman N. Vermeersch P. Heijboer A. C. (2024). Vitamin D metabolites before and after kidney transplantation in patients who are anephric. Am. J. Kidney Dis. 84, 427–436.e1. 10.1053/j.ajkd.2024.03.025 38796137

[B9] Kido K. Wheeler M. B. Seratnahaei A. Bailey A. Bain J. A. (2015). Rhabdomyolysis precipitated by possible interaction of ticagrelor with high-dose atorvastatin. J. Am. Pharm. Assoc. (2003) 55, 320–323. 10.1331/JAPhA.2015.14151 26003161

[B10] Kunakorntham P. Pattanaprateep O. Dejthevaporn C. Thammasudjarit R. Thakkinstian A. (2022). Detection of statin-induced rhabdomyolysis and muscular related adverse events through data mining technique. BMC Med. Inf. Decis. Mak. 22, 233. 10.1186/s12911-022-01978-4 36064346 PMC9446837

[B11] Maheta D. Agrawal S. P. Patel J. Patel M. Frishman W. H. Aronow W. S. (2025). Curbside consult optimizing Statin therapy in the elderly: a personalized approach to cardiovascular disease prevention. Cardiol. Rev. 10.1097/CRD.0000000000000848 39750022

[B12] Martin G. L. Létinier L. (2026). Artificial intelligence in personalized prescription: a narrative review of promise, peril, and practicality. Therapie 81, 132–139. 10.1016/j.therap.2025.10.001 41966058

[B13] Migliorini F. Maffulli N. Eschweiler J. Baroncini A. Bell A. Colarossi G. (2023). Duloxetine for fibromyalgia syndrome: a systematic review and meta-analysis. J. Orthop. Surg. Res. 18, 504. 10.1186/s13018-023-03995-z 37461044 PMC10351165

[B14] Mummadi S. R. Mishra R. (2018). Effectiveness of provider price display in computerized physician order entry (CPOE) on healthcare quality: a systematic review. J. Am. Med. Inf. Assoc. 25, 1228–1239. 10.1093/jamia/ocy076 29982523 PMC7646898

[B15] Nature M. (2025). For trustworthy AI, keep the human in the loop. Nat. Med. 31, 3207. 10.1038/s41591-025-04033-7 41073516

[B16] Olawade D. B. Plabon S. B. Ojo A. Ogunbona M. A. Makanjuola B. D. Olasilola O. R. (2026). Human in the loop artificial intelligence in healthcare: applications, outcomes, and implementation challenges. Int. J. Med. Inf. 213, 106362. 10.1016/j.ijmedinf.2026.106362 41740273

[B17] Ong J. C. L. Jin L. Elangovan K. Lim G. Y. S. Lim D. Y. Z. Sng G. G. R. (2025). Large language model as clinical decision support system augments medication safety in 16 clinical specialties. Cell Rep. Med. 6, 102323. 10.1016/j.xcrm.2025.102323 40997804 PMC12629785

[B18] Ong A. Y. Merle D. A. Pollreisz A. Wagner S. K. Sevgi M. Keane P. A. (2026). Flight rules for clinical AI: lessons from aviation for human-AI collaboration in medicine. NPJ Digit. Med. 9, 201. 10.1038/s41746-026-02410-1 41620563 PMC12963479

[B19] Pedersen T. R. (2010). Pleiotropic effects of statins: evidence against benefits beyond LDL-Cholesterol lowering. Am. J. Cardiovasc Drugs 10 (Suppl. 1), 10–17. 10.2165/1158822-S0-000000000-00000 21391729

[B20] Rubino J. MacDougall D. E. Sterling L. R. Hanselman J. C. Nicholls S. J. (2021). Combination of bempedoic acid, ezetimibe, and atorvastatin in patients with hypercholesterolemia: a randomized clinical trial. Atherosclerosis 320, 122–128. 10.1016/j.atherosclerosis.2020.12.023 33514449

[B21] Şahan E. Parlakkaya Yıldız F. B. (2019). Duloxetine induced hyponatremia. Turk Psikiyatri Derg. 30, 287–289. 10.4103/0971-4065.151352 32594491

[B22] Sousa V. T. D. S. Fernandes E. da S. Costa E. C. Moura J. R. S. A. de Melo E. S. J. Carvalho R. E. F. L. de (2025). 360° video simulation scenario for the WHO global patient safety challenge “Medication Without Harm.”. Nurse Educ. 50, E31–E36. 10.1097/NNE.0000000000001738 39288339

[B23] Spanakis M. De Pauw A. Brumer M. Symvoulakis E. K. De Loof H. (2026). Closing the loop: human-augmented, mechanistically enhanced AI for proactive management of drug-drug interactions. Front. Pharmacol. 17, 1767646. 10.3389/fphar.2026.1767646 41958940 PMC13057478

[B24] Stone N. J. Robinson J. G. Lichtenstein A. H. Bairey Merz C. N. Blum C. B. Eckel R. H. (2014). 2013 ACC/AHA guideline on the treatment of blood cholesterol to reduce atherosclerotic cardiovascular risk in adults: a report of the American college of cardiology/American heart association task force on practice guidelines. J. Am. Coll. Cardiol. 63, 2889–2934. 10.1016/j.jacc.2013.11.002 24239923

[B25] Tantray J. Patel A. Wani S. N. Kosey S. Prajapati B. G. (2024). Prescription precision: a comprehensive review of intelligent prescription systems. Curr. Pharm. Des. 30, 2671–2684. 10.2174/0113816128321623240719104337 39092640

[B26] Yee C. S. Hawken E. R. Baldessarini R. J. Vázquez G. H. (2019). Maintenance pharmacological treatment of juvenile bipolar disorder: review and meta-analyses. Int. J. Neuropsychopharmacol. 22, 531–540. 10.1093/ijnp/pyz034 31211354 PMC6672626

[B27] Zhang M. Ma W.-C. He W.-Y. (2025). Artificial intelligence-assisted quality control circles led by clinical pharmacists to improve the rational use of parenteral proton pump inhibitors among hospitalised patients. Sci. Rep. 15, 31391. 10.1038/s41598-025-16934-1 40858969 PMC12381210

