# Supplementary material for: Descriptive analysis of prescription interception patterns: characterizing medication safety risks in an outpatient setting
Source: Front Pharmacol. 2026 Jul 14;17:1826012. doi: 10.3389/fphar.2026.1826012 (PMC13408375; doi:10.3389/fphar.2026.1826012)
Supplement: Supplementary file 1 [file Table1.docx]

**STable 1 Departments' FI Prescriptions Specific Situations**

| **Department** |  |  | **Count** | **Proportion** |
| --- | --- | --- | --- | --- |
| Department of Mental Health | Issues | Medication beyond the treatment course | 4119 | 72.19% |
|  |  | Medication beyond the prescribed dosage | 480 | 8.41% |
|  | Drugs | Duloxetine Hydrochloride Enteric-coated Capsules | 1770 | 31.02% |
|  |  | Tandospirone Citrate Capsules | 1444 | 25.31% |
|  |  | Bupropion Hydrochloride Sustained-release Tablets | 755 | 13.23% |
|  | Diagnosis | Anxiety and depression state | 4458 | 78.13% |
|  |  | Mental disorder | 557 | 9.76% |
|  |  | Sleep disorder | 426 | 7.47% |
| Department of Neurology | Issues | Medication beyond the treatment course | 3479 | 62.25% |
|  |  | Medication beyond the prescribed dosage | 1377 | 24.64% |
|  | Drugs | Pregabalin Capsules | 277 | 4.96% |
|  |  | Alendronate Sodium Tablets | 247 | 4.42% |
|  |  | Pyridostigmine Bromide Tablets | 246 | 4.40% |
|  | Diagnosis | Myasthenia gravis | 1036 | 18.54% |
|  |  | Insomnia | 955 | 17.09% |
|  |  | Osteoporosis | 702 | 12.56% |
| Department of Nephrology | Issues | Medication beyond the treatment course | 3107 | 58.60% |
|  |  | Medication beyond the prescribed dosage | 297 | 5.60% |
|  | Drugs | Atorvastatin Calcium Tablets | 328 | 8.38% |
|  |  | Vitamin D Drops | 304 | 7.77% |
|  |  | Shenyanling Granules | 236 | 6.03% |
|  | Diagnosis | Nephrotic syndrome | 1047 | 26.75% |
|  |  | Hypertension | 1009 | 25.78% |
|  |  | Renal insufficiency | 648 | 16.56% |
| Department of Cardiovascular Medicine | Issues | Medication beyond the treatment course | 2265 | 74.3% |
|  |  | Medication beyond the prescribed dosage | 364 | 11.90% |
|  | Drugs | Clopidogrel tablets | 667 | 21.88% |
|  |  | Atorvastatin Calcium Tablets | 475 | 15.60% |
|  |  | Dapagliflozin tablets | 182 | 6.00% |
|  | Diagnosis | Hyperlipidemia | 1433 | 47.31% |
|  |  | Hypertension | 913 | 30.14% |
|  |  | Coronary atherosclerotic heart disease | 571 | 18.85% |
| Department of Endocrinology and Metabolism | Issues | Medication beyond the treatment course | 1758 | 72.76% |
|  |  | Medication beyond the prescribed dosage | 264 | 10.93% |
|  | Drugs | Atorvastatin Calcium Tablets | 318 | 13.16% |
|  |  | Insulin Degludec and Insulin Aspart Injection | 258 | 10.68% |
|  |  | Sitagliptin and Metformin Tablets | 175 | 7.24% |
|  | Diagnosis | Diabetes mellitus | 1476 | 61.09% |
|  |  | Hyperlipidemia | 743 | 30.75% |
|  |  | Hypertension | 356 | 14.74% |

**STable 2 The proportions of IF Prescriptions by Departments**

| Department | IF | SUM | Proportion（%） |
| --- | --- | --- | --- |
| Department of Mental Health | 5706 | 79465 | 7.18% |
| Department of Neurology | 5589 | 102156 | 5.47% |
| Department of Nephrology | 2459 | 23325 | 10.54% |
| Department of Cardiovascular Medicine | 2868 | 48437 | 5.92% |
| Department of Endocrinology and Metabolism | 2416 | 39301 | 6.15% |
